# Hemolymph metabolism of black soldier fly (Diptera: Stratiomyidae), response to different supplemental fungi

**DOI:** 10.1093/jisesa/ieae050

**Published:** 2024-05-07

**Authors:** Mani Kannan, Tzach Vitenberg, Ron Schweitzer, Itai Opatovsky

**Affiliations:** Laboratory of Insect Nutrition and Metabolism, Department of Nutrition and Natural Products, MIGAL-Galilee Research Institute, Kiryat Shmona, Israel; Department of Animal Science, Faculty of Sciences and Technology, Tel-Hai College, 11 Upper Galilee, Israel; Laboratory of Insect Nutrition and Metabolism, Department of Nutrition and Natural Products, MIGAL-Galilee Research Institute, Kiryat Shmona, Israel; Department of Natural Compounds and Analytical Chemistry, MIGAL-Galilee Research Institute, Kiryat Shmona, Israel; Laboratory of Insect Nutrition and Metabolism, Department of Nutrition and Natural Products, MIGAL-Galilee Research Institute, Kiryat Shmona, Israel; Department of Animal Science, Faculty of Sciences and Technology, Tel-Hai College, 11 Upper Galilee, Israel

**Keywords:** fungal supplementation, larval development, hemolymph metabolite, metabolomic analysis

## Abstract

The black soldier fly, *Hermetia illucens* L. (Diptera: Stratiomyidae), is commonly used for organic waste recycling and animal feed production. However, the often inadequate nutrients in organic waste necessitate nutritional enhancement of black soldier fly larvae, e.g., by fungal supplementation of its diet. We investigated the amino acid composition of two fungi, *Candida tropicalis* (Castell.) Berkhout (Saccharomycetales: Saccharomycetaceae) and *Pichia kudriavzevii* Boidin, Pignal & Besson (Saccharomycetales: Pichiaceae), from the black soldier fly gut, and commercial baker’s yeast, *Saccharomyces cerevisiae* Meyen *ex* E.C. Hansen (Saccharomycetales: Saccharomycetaceae), and their effects on larval growth and hemolymph metabolites in fifth-instar black soldier fly larvae. Liquid chromatography–mass spectrometry was used to study the effect of fungal metabolites on black soldier fly larval metabolism. Amino acid analysis revealed significant variation among the fungi. Fungal supplementation led to increased larval body mass and differential metabolite accumulation. The three fungal species caused distinct metabolic changes, with each over-accumulating and down-accumulating various metabolites. We identified significant alteration of histidine metabolism, aminoacyl-tRNA biosynthesis, and glycerophospholipid metabolism in BSF larvae treated with *C. tropicalis*. Treatment with *P. kudriavzevii* affected histidine metabolism and citrate cycle metabolites, while both *P. kudriavzevii* and *S. cerevisiae* treatments impacted tyrosine metabolism. Treatment with *S. cerevisiae* resulted in down-accumulation of metabolites related to glycine, serine, and threonine metabolism. This study suggests that adding fungi to the larval diet significantly affects black soldier fly larval metabolomics. Further research is needed to understand how individual amino acids and their metabolites contributed by fungi affect black soldier fly larval physiology, growth, and development, to elucidate the interaction between fungal nutrients and black soldier fly physiology.

## Graphical Abstract

**Graphical Abstract:**
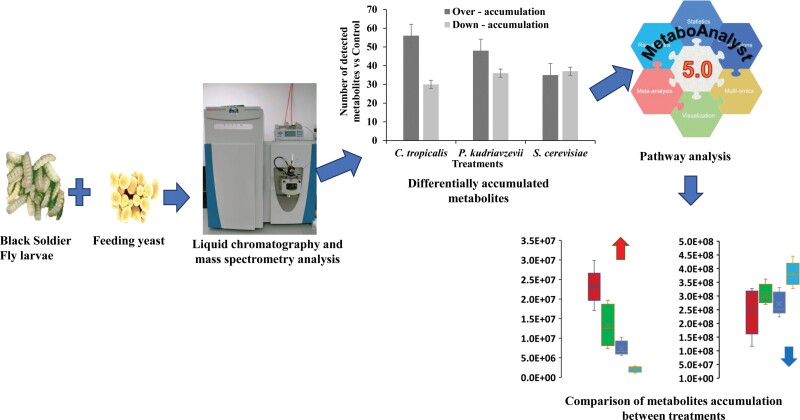

## Introduction

The increasing global human population, coupled with a rising demand for animal meat, imposes a significant burden on the food industry. Due to the scarcity and high cost of feed, it is difficult to produce the required quantities of meat; therefore, there is a need for alternative protein sources. One of the sustainable alternatives is the mass production of insects as a source of protein (van Huis and Oonincx, 2017), e.g., crickets, mealworms, etc. (Ortiz et al. 2016). In this regard, the black soldier fly, *Hermetia illucens* (L.), which has received approval from the EU for use as animal feed in the livestock and aquaculture industries only (not for human consumption), is one of the main insects being reared to address the high costs associated with animal feed production (Bosch et al. 2019, Kawasaki et al. 2019, Smetana et al. 2019, Mouithys-Mickalad et al. 2020). The black soldier fly may be reared on various organic wastes, such as food waste, animal waste and manure, bread, industrial residue, and agricultural waste, thereby ensuring cost-effective and environmentally sustainable rearing practices (Rehman et al. 2017, Kim et al. 2021). Several companies have emerged in different countries, including Israel, the United Kingdom, the United States, and the Netherlands, and are actively involved in mass-rearing and industrial-scale production of the black soldier fly. Employing advanced techniques such as artificial intelligence and modified fly genomics, these companies aim to optimize and expand black soldier fly production while enhancing its nutritional profile (Gruber and Melton 2023). Furthermore, these enterprises are dedicated to supplying insect factories with ready-to-use suspended neonates and frozen eggs, supporting the production of industrial-scale animal feed for livestock, aquaculture, and the pet industry (Barragan-Fonseca et al. 2017, Chia et al. 2020, Hall et al. 2021, Gruber and Melton 2023).

Black soldier fly larvae also play a crucial role in waste management by recycling solid waste and reducing pathogen loads in waste compost (Lalander et al. 2013, 2015, Chia et al. 2018, Isibika et al. 2019, Wynants et al. 2019, Mazza et al. 2020, Borel et al. 2021, Salam et al. 2021). They turn organic waste into compost that can be used as fertilizer to enhance the growth of vegetable crops (Anyega et al. 2021, Chiam et al. 2021). Furthermore, the lipids derived from the black soldier fly have been recognized as a valuable source for biodiesel production (Wang et al. 2017, Ishak et al. 2018, Ishak and Kamari 2019, Lee et al. 2021, Park et al. 2021, Jung et al. 2022, Liew et al. 2022). Moreover, the black soldier fly larva produces antimicrobial peptides effective against bacteria, fungi, and viruses (Vogel et al. 2018, Alvarez et al. 2019, Moretta et al. 2020, Xia et al. 2021, van Moll et al. 2022).

To enhance the body weight and nutritional value of black soldier fly larvae, research to date has focused on feeding them with different organic substrates supplemented with microorganisms such as bacteria, algae, and fungi (Yu et al. 2011, Kooienga et al. 2020, Franks et al. 2021, Elhag et al. 2022, Waters 2022). For instance, the addition of bacteria to the black soldier fly diet or organic waste such as chicken manure has been shown to improve the conversion of organic waste, reduce the transmission of waste-borne pathogens, and increase larval biomass, protein, and lipid content (Yu et al. 2011, Kooienga et al. 2020, Wong et al. 2020, 2021b, Franks et al. 2021, Elhag et al. 2022, Waters 2022). Interestingly, the supplementation of algae, either alone (e.g., *Schizochytrium spp*.) or in combination with brewer’s spent grain (a byproduct of malted barley grains generated during the beer brewing process), has been found to promote larval weight gain and the accumulation of omega-3 fatty acids (El-Dakar et al. 2020, Ceccotti et al. 2022).

Similarly, supplementing fungi with agro-industrial products, such as molasses, has been found to enhance black soldier fly larval weight, protein, and lipid content (Chia et al. 2018, 2020). Studies have shown that agricultural byproducts, such as cacao pod husks and oil palm fronds, inoculated with fungi, such as *Phanerochaete chrysosporium*, *Trametes versicolor*, and *Pleurotus sajor*, promote waste reduction and increase black soldier fly fatty acid and protein levels, including glutamic and leucine amino acid composition (Fitriana et al. 2022). Other studies have demonstrated that black soldier fly larvae can attain higher lipid and protein content when fed substrates containing fermented coconut endosperm waste with yeasts (baker’s yeast) or fungi (*Rhizopus oligosporus*), or nonfermented palm kernel expeller with fungi (Wong et al. 2020, 2021a, 2021b, Liew et al. 2022). For example, the combination of *Candida lipolytica* CL2 with black soldier fly larvae offers a synergistic approach to waste oil treatment. The yeast helps to reduce the fat content of the oil, while the larvae efficiently consume the two and convert them into valuable fatty acids, contributing to the overall recycling and valorization of waste resources (Lu et al. 2023).

The present study proposed the use of microorganisms, specifically fungi isolated from the insect’s gut, as they are better adapted to the insect’s nutritional system and will have a greater effect on the insect’s body condition and nutritional composition. Recent studies on dietary supplementation with *C. tropicalis*, a yeast-like fungus from the black soldier fly gut known to contain over 50% protein and 5% lipids, have demonstrated an increase in larval body weight and alteration of whole-body metabolites compared to supplementation with *Saccharomyces cerevisiae* (baker’s yeast, not commonly found in the black soldier fly gut) (Mani et al. 2023a). The metabolomic profile of the entire black soldier fly body is affected by larval digestion of the yeast-supplemented diet. However, it is unclear which metabolites are released from the gut into the hemolymph, which functions as a nutritional reservoir, transporting nutrients to various tissues to support insect growth and metamorphosis (Kannan et al. 2016). Therefore, in this study, we tested supplementation with different fungal species that were isolated from the black soldier fly gut to determine whether specific metabolites are absorbed by the gut and released into the hemolymph. In addition, we investigated amino acid transfer from the fungi to the black soldier fly and analyzed the amino acid composition of the fungi to determine whether this altered amino acid metabolism, as shown in a previous study (Mani et al. 2023a). A study of the metabolites in the hemolymph could shed light on physiological changes related to fungal metabolism in the black soldier fly larval gut and provide insights regarding the promising use of fungi as dietary supplements for black soldier fly. This study could provide a way to enhance black soldier fly larval growth and weight for animal feed production, emphasizing the improved suitability of black soldier fly larvae after fungal supplementation.

## Materials and Methods

### Fungal Culture Preparation for Amino Acid Analysis

Pure isolates of the fungi, *Candida tropicalis* (Castell.) Berkhout, *Pichia kudriavzevii* Boidin, Pignal & Besson, and baker’s yeast (*S. cerevisiae* Meyen *ex* E.C. Hansen) were subcultured in yeast extract peptone dextrose (YPD) broth treated with chloramphenicol (1 µl/ml, stock: 34 mg/ml). The subcultured fungi were reintroduced (1%) into 700 ml of chloramphenicol-treated YPD broth. The fungal cultures were grown for 48 h at 28 °C. The fungal cells were then harvested by centrifugation at 4 °C for 3 min. The supernatant was discarded, and the pellet was washed twice with Milli-Q water and frozen at −80 °C. The frozen pellet was subjected to freeze-drying, which involved freezing at a temperature below −40 °C followed by controlled drying under vacuum. The freeze-dried fungi were stored at 4 °C. The dried fungal samples were sent to the Molecular Structure Facility, University of California, Davis, CA, USA, for amino acid analysis, performed as follows: approximately 4 mg for *P. kudriavzevii*, 4.5–4.6 mg for *S. cerevisiae*, and 4.6–4.7 mg for *C. tropicalis* of dried fungus were transferred to a hydrolysis tube and subjected to liquid-phase hydrolysis using phenolic-hydrochloric acid (200 µl of 6 normality HCl/1% phenol) at 110 °C for 24 h. The sample was then dried. The hydrolyzed sample with an internal standard was vortexed and spun down, and 50 µl of the solution was injected into the Hitachi L-8900 (Li-based) analyzer (Tokyo, Japan). Nor-leucine was added as the internal standard with a final concentration of 2 nM/5 µl of injection. Quantification of each amino acid was performed by determining its concentration relative to an internal standard, and the results were expressed as a percentage using the calculation provided in the UCDavis portal (https://msf.sf.ucdavis.edu/amino-acid-analysis-calculations). The raw data have been included in Supplementary File 1.

### Black Soldier Fly Diet Preparation and Feeding Experiments

Following the protocol of Mani et al. (2023a), approximately 5 g (wet weight) of fresh fungal pellet for each fungal treatment was added to a sawdust diet. Five-day-old black soldier fly larvae were released into a rearing flask for each treatment (T1: water as control (W), no fungi, T2: *C. tropicalis*, T3: *P. kudriavzevii*, and T4: *S. cerevisiae*), with 5 replicates per treatment. In the control treatment, 0.625 g casein was added as a protein source, equivalent to 5 g yeast fungus by wet weight. The larvae were allowed to mature until they entered the pupal stage, which was distinguished by a black coloration. This stage was considered achieved when at least 10% of the larvae exhibited a distinct black hue. We note that individual larval sizes or stages may vary slightly within the larval pool. To ensure a consistent larval stage for body weight measurement and hemolymph sample collection in the metabolomics study, we waited until pupation in at least 10% of the larvae. The hemolymph was collected from final instar larvae into 1.7-ml Eppendorf tubes containing 10 µl of phenylthiourea (50 mg/ml), following a previously described method (Kannan et al. 2016). The collected hemolymph was immediately centrifuged at 13,000 rpm, at 4 °C for 10 min to remove cells and tissue debris. The clear supernatant of the hemolymph was used for metabolite extraction with slight modifications, as previously reported (Zhou et al. 2021). In brief, 100 µl of the hemolymph was mixed with 700 µl acidic methanol (80% methanol with 0.1% formic acid), vortexed, and centrifuged at 13,000 rpm, at 4 °C for 20 min. For the experimental blank, 100 µl acidic methanol was used instead of hemolymph and mixed with 700 µl acidic methanol. The same procedure was followed for hemolymph metabolite extraction All the extracts were then filtered using a membrane filter with a pore size of 0.22 µm. The filtered extracts were used for quality control (QC), preparation by pooling equal volumes of different samples, and LC–MS (liquid chromatography–mass spectrometry) analysis to normalize the data. The detailed methods for LC conditions for metabolite separation, MS parameters for metabolite detection, metabolite annotation, and other relevant procedures were performed as described in Mani et al. (2023b).

### Metabolic Pathway Analysis

To visually describe the differences in metabolic patterns and clustering results between different groups and identify the impactful pathways associated with the annotated metabolites from the different fungal treatments compared to the water treatment, principal component analysis and pathway enrichment analysis, respectively, were conducted using MetaboAnalyst 5.0 (Pang et al. 2021). This software tool is equipped with a comprehensive library of metabolic pathways, using KEGG (Kyoto Encyclopedia of Genes and Genomes) identifiers to facilitate the annotation of metabolites in different metabolic pathways. During pathway enrichment analysis, the following parameters were applied: the model organism library selected was *Drosophila melanogaster*, and statistical comparisons were performed using the *hypergeometric test*. The pathways that exhibited significant effects were determined based on their respective *P*-values, following the approach described by Zhang et al. (2022). Some of the metabolites (pyridoxal, valine, quinolinic acid, arginine, hypoxanthine, and citric acid) belonging to different pathways were not enriched during pathway analysis. However, their patterns were consistent with those observed in a previous study (Mani et al. 2023a).

### Data Analysis

The significant difference in larval body weight between the treatments (control and fungi) was analyzed using 1-way ANOVA followed by Tukey’s pairwise comparison. To assess significant differences in the normalized intensity peak area of metabolites among the different fungal treatments, 1-way ANOVA and Tukey’s pairwise comparisons were performed.

## Results

### Amino Acid Composition of the Different Fungi

The amino acid analysis revealed that *C. tropicalis* (Ct), *P. kudriavzevii* (Pk), and *S. cerevisiae* (Sc) had protein contents of 47.56%, 44.06%, and 42.31%, respectively, with similar amino acid composition (Table 1). Although not significant, some essential amino acids showed small differences between the fungal species. Arginine was higher in *S. cerevisiae* (3.1 ± 0.1%) compared to *P. kudriavzevii* and *C. tropicalis* (2.8% ± 0.1% and 2.8% ± 0.1%, respectively), while histidine was higher in *P. kudriavzevii* (1.3% ± 0.1%) compared to *S. cerevisiae* and *C. tropicalis* (1.2% ± 0.1% and 1.0% ± 0.1%, respectively). Similarly, leucine, lysine, phenylalanine, and threonine were higher in *P. kudriavzevii* compared to the other 2 fungi (leucine: Pk—3.9% ± 0.1%, Sc—3.5% ± 0.1%, and Ct—3.4% ± 0.3%; lysine: Pk—4.8% ± 0.1%, Sc—4.5% ± 0.1%, and Ct—4.3% ± 0.2%; phenylalanine: Pk—2.4% ± 0.1%, Sc—2.1% ± 0.1%, and Ct—2.0% ± 0.1%; threonine: Pk—2.7% ± 0.0%, Sc—2.30% ± 0.0%, and Ct—2.3% ± 0.1%). The percentages of isoleucine and valine were found to be the same among the fungi. Among the nonessential amino acids, asparagine, or aspartic acid and tyrosine were higher in *P. kudriavzevii* compared to *S. cerevisiae* and *C. tropicalis* (asparagine: Pk—5.6% ± 0.1%, Sc—4.8% ± 0.1%, and Ct—4.6% ± 0.1%; tyrosine: Pk—1.8% ± 0.1%, Sc—1.6% ± 0.1%, and Ct—1.6% ± 0.1%). Glycine was higher in *S. cerevisiae* than in *P. kudriavzevii* and *C. tropicalis* (Sc—3.1% ± 0.1%, Pk—2.8% ± 0.1%, and Ct—2.6% ± 0.0%). The average percentages of these specific amino acids (alanine, proline, glutamine, or glutamic acid and serine) were found to be similar across different fungi.

**Table 1.** Percentage of amino acids in fungal dry weight

Name of the amino acid	Amino acids (average of percentage ± SE) in different yeasts		
	*Pichia kudriavzevii*	*Saccharomyces cerevisiae*	*Candida tropicalis*
Arginine	2.8 ± 0.1	3.1 ± 0.1	2.8 ± 0.1
Histidine	1.3 ± 0.1	1.2 ± 0.1	1.0 ± 0.1
Isoleucine	2.7 ± 0.1	2.4 ± 0.1	2.4 ± 0.2
Leucine	3.9 ± 0.1	3.5 ± 0.1	3.4 ± 0.2
Lysine	4.8 ± 0.1	4.5 ± 0.1	4.3 ± 0.2
Phenylalanine	2.4 ± 0.1	2.1 ± 0.1	2.0 ± 0.1
Threonine	2.7 ± 0.1	2.3 ± 0.1	2.3 ± 0.1
Valine	2.9 ± 0.1	2.6 ± 0.1	2.6 ± 0.1
Alanine	3.1 ± 0.1	2.9 ± 0.1	3.0 ± 0.1
Aspartate or asparagine	5.6 ± 0.1	4.8 ± 0.1	4.6 ± 0.1
Glycine	2.8 ± 0.1	3.1 ± 0.1	2.6 ± 0.1
Glutamate or glutamine	6.3 ± 0.1	5.6 ± 0.5	5.3 ± 0.2
Tyrosine	1.8 ± 0.1	1.6 ± 0.1	1.6 ± 0.1
Proline	2.2 ± 0.1	2.2 ± 0.1	2.2 ± 0.2
Serine	2.3 ± 0.1	2.2 ± 0.1	2.2 ± 0.1

Approximately 4 mg of fungi was used as 100%. Two replicates were used for amino acid quantification for each fungal species.

### Analysis of Black Soldier Fly Larval Body Weight Following Fungal Supplementation

**Fig. 1.**
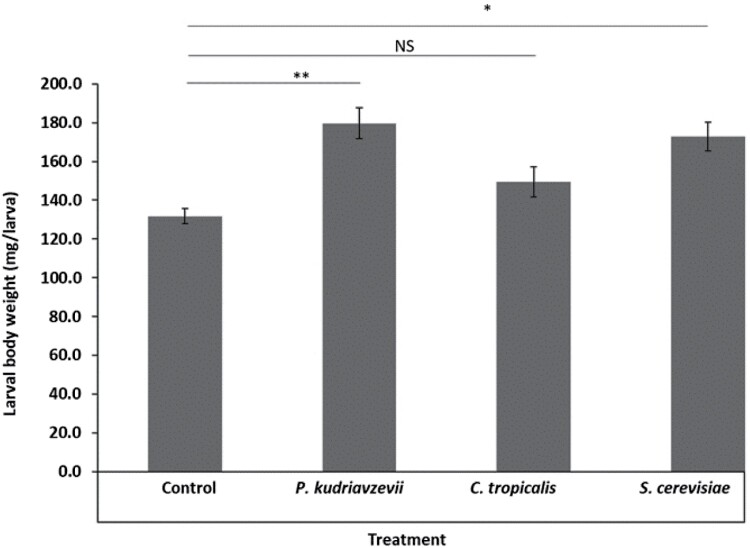
Effect of feeding with fungal supplements on average larval body weight (milligram) of the black soldier fly. The error bars represent SE. Control (no fungus), *P. kudriavzevii*—*Pichia kudriavzevii*, *C. tropicalis*—*Candida tropicalis*, and *S. cerevisiae*—*Saccharomyces cerevisiae*. The asterisk above the line between the control and different fungi indicates the level of significance, *P < 0.005, **P < 0.001, and NS, nonsignificant.

The larvae that were supplemented with *P. kudriavzevii* and *S. cerevisiae* (but not *C. tropicallis*) had higher body weight (179.6 ± 8.1 and 172.8 ± 7.3 mg, respectively) than the control (131.8 ± 3.9 mg) (*P* < 0.001; Fig. 1).

### Principal Component Analysis of Detected Metabolites

Metabolomic analysis of the black soldier fly larva hemolymph resulted in the detection of 701 metabolites in total, which were subsequently normalized with QC (relative standard deviation, RSD < 50%). Of these, 464 metabolites were successfully annotated with identified compound names. Tandem mass spectroscopy further annotated 190 metabolites, with 122 having >80% fragment similarity and 68 having 50%–80% fragment similarity. The remaining metabolites were identified using the ChemSpider database through isotope abundance and mass accuracy.

**Fig. 2.**
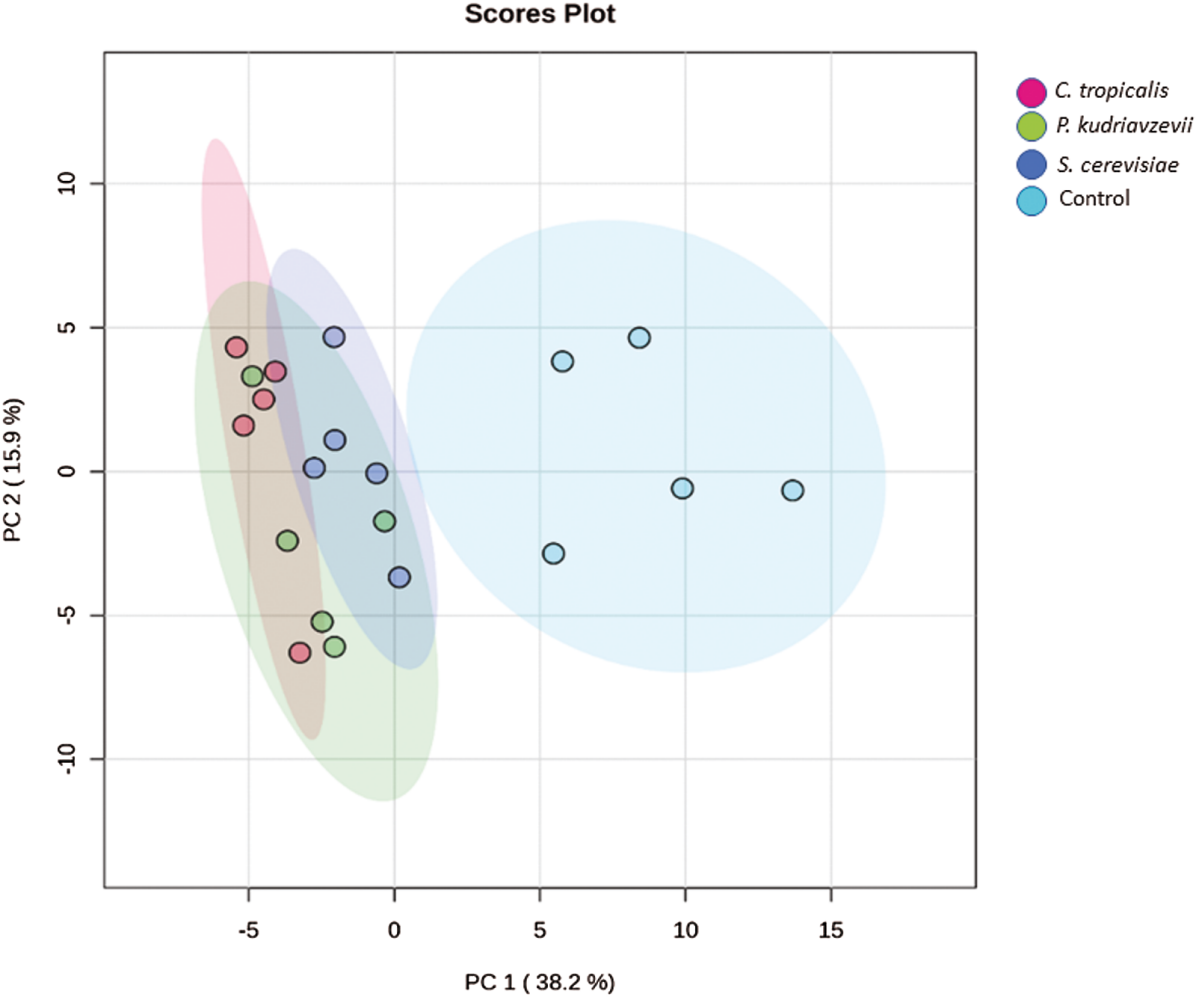
Principal components analysis scores for total metabolites from 4 treatment groups. *C. tropicalis*—*Candida tropicalis*, *P. kudriavzevii*—*Pichia kudriavzevii*, *S. cerevisiae*—*Saccharomyces cerevisiae*, and Control (no fungus). The *x*-axis and *y*-axis represent variance (PC1: 38.2% and PC2: 15.9%) among treatments, respectively. Five replicates were used per treatment.

To evaluate significant changes in the overall metabolite profiles among the treatments, principal component analysis (PCA) was performed (Fig. 2). The PCA plot clearly demonstrates distinct separations between the water group and the groups treated with fungi, indicating that fungi have a discernible impact on the metabolite composition of black soldier fly larvae. Additionally, the close clustering of the 5 biological replicates within each group suggests good homogeneity among replicates and high data reliability. The PCA accounted for 54.1% of the total variance, indicating that the observed variation in metabolite compositions is highly informative. The similarities observed in metabolite compositions between *P. kudriavzevii*, *S. cerevisiae*, and *C. tropicalis* imply shared fungal metabolism within the gut of black soldier fly larvae.

### Analysis of the Differential Regulation of Metabolites

**Fig. 3.**
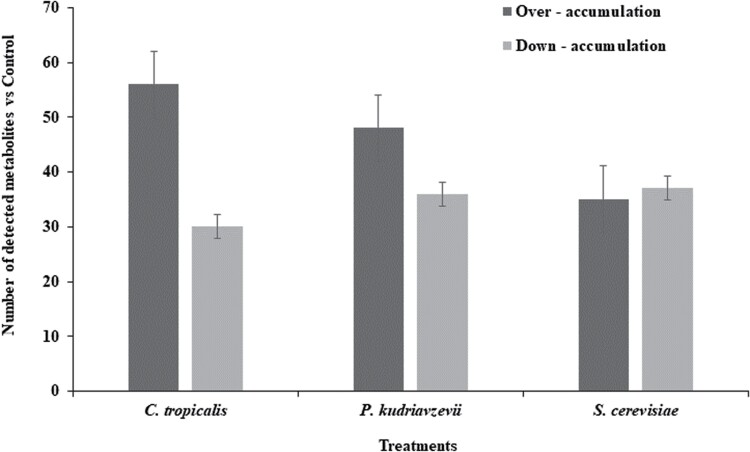
Differential accumulation of metabolites in fungi compared to the control group (no fungus added). The bars represent the mean, and the error bars represent the SEM for each treatment. *C. tropicalis*—*Candida tropicalis*, *P. kudriavzevii*—*Pichia kudriavzevii*, and *S. cerevisiae*—*Saccharomyces cerevisiae*.

The hemolymph metabolome of black soldier fly larvae from the 3 yeast treatments and the observed variation in the accumulation of metabolites were compared to the water treatment. Specifically, we found that *C. tropicalis* had 56 over-accumulated metabolites and 30 down-accumulated metabolites; *P. kudriavzevii* had 48 over-accumulated metabolites and 36 down-accumulated metabolites; and *S. cerevisiae* had 35 over-accumulated metabolites and 37 down-accumulated metabolites (Fig. 3). The classification of the metabolites for *C. tropicalis*, *P. kudriavzevii*, and *S. cerevisiae* compared to the water treatment is presented in Supplementary Tables S1–S3, respectively.

### Pathway Enrichment Analysis to Identify the Pathways Affected After Feeding With Fungi

**Fig. 4.**
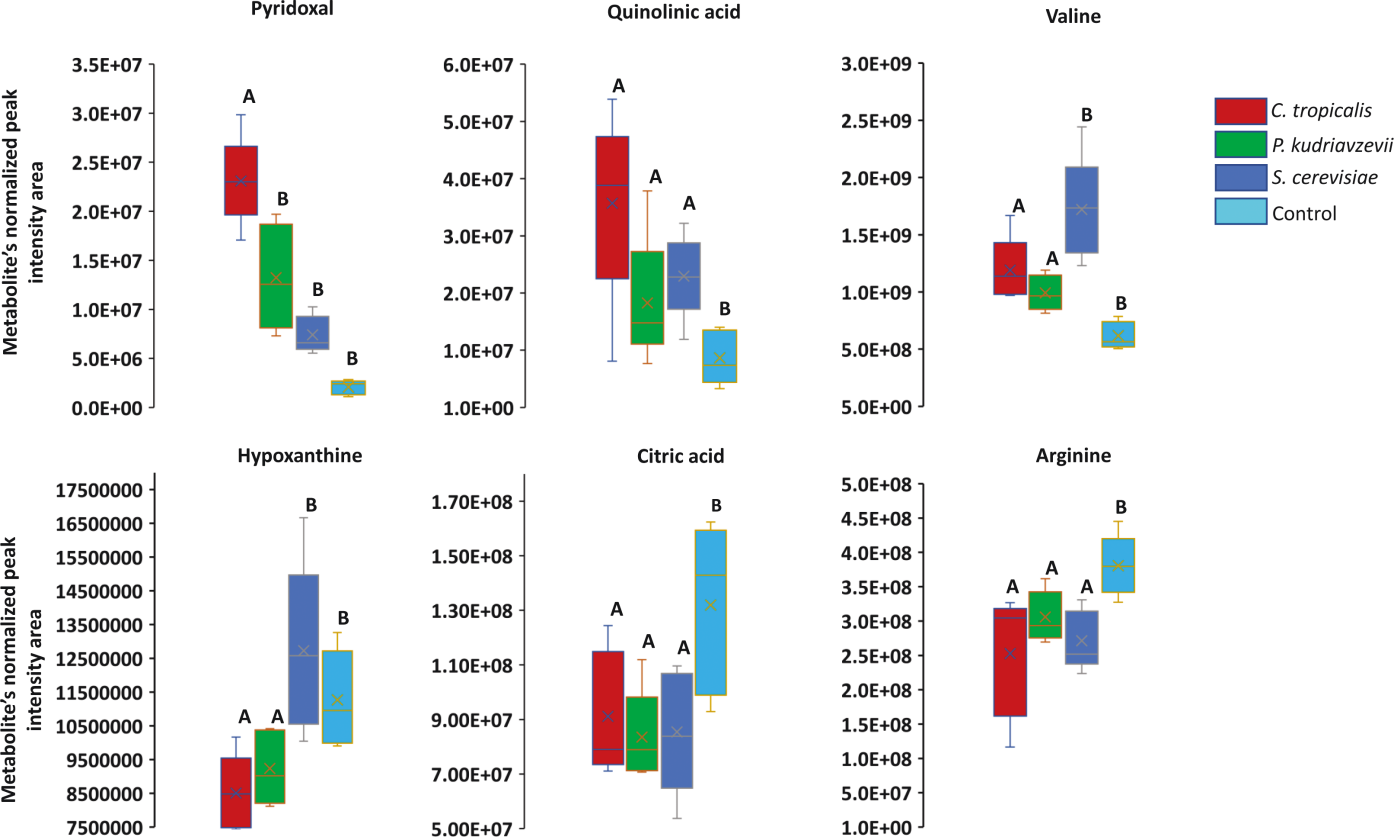
The box plot of normalized peak intensity area of differentially accumulated metabolites with nonenriched metabolic pathways. Different letters above the box plot indicate a statistically significant difference (*P* < 0.05) between the control and each fungus by Tukey’s pairwise comparisons following 1-way ANOVA.

Supplementation with different fungi affected different metabolic pathways in black soldier fly larvae (Table 2). The larvae supplemented with *C. tropicalis* showed an increase in metabolites related to histidine metabolism, aminoacyl-tRNA biosynthesis, and glycerophospholipid metabolism. Similarly, the larvae supplemented with *P. kudriavzevii* exhibited an increase in metabolites related to histidine metabolism, as well as tyrosine metabolism and the citrate cycle. Finally, the larvae supplemented with *S. cerevisiae* showed an increase in metabolites related to glycine, serine, and threonine metabolism, as well as tyrosine metabolism. Some metabolites showed significant over- and down-accumulation in each fungal treatment compared to the water treatment (W) but did not affect the whole metabolic pathway. Specifically, pyridoxal (*P* < 0.001), a metabolite of B6-vitamin, quinolinic acid (*P* = 0.01), derived from tryptophan, and valine (*P* < 0.001), a major metabolite from the valine, leucine, and isoleucine biosynthesis pathway, were significantly over-accumulated in all fungal treatments compared to the control. Conversely, hypoxanthine (*P* = 0.003), involved in purine metabolism; citric acid (*P* = 0.01), related to alanine, aspartate, and glutamate metabolism; and arginine (*P* = 0.003), related to arginine synthesis, were significantly down-accumulated in all 3 fungal treatments compared to the control (Fig. 4).

**Table 2.** Differential effects of fungi on metabolic pathways in the larval hemolymph of *Hermetia illucens*, compared to the control

Impactful metabolic pathways	Name of all detected and annotated metabolites from hemolymph in each pathway	Number of detected/annotated metabolites out of total metabolites in each pathway	Name of significantly altered metabolites in each pathway^a^	*P*-value	Pathway direction
*Candida tropicalis*					
Histidine metabolism	l-Histidine; imidazole-4-acetate; methylimidazoleacetic acid	3/9	Imidazoleacetic acid, l-histidine	0.002	Down
Aminoacyl-tRNA biosynthesis	l-Asparagine; l-histidine; l-phenylalanine; l-arginine; l-glutamine; glycine; l-serine; l-valine; l-lysine; l-isoleucine; l-threonine; l-tyrosine; l-proline; l-glutamate	14/48	**Valine**, l-arginine, l-histidine	0.005	Both up and down
Glycerophospholipid metabolism	1-Acyl-sn-glycero-3-phosphocholine; choline phosphate; acetylcholine; 1-acyl-sn-glycero-3-phosphoethanolamine	4/32	**Acetylcholine, phosphocholine**	0.027	Up
*Pichia kudriavzevii*					
Histidine metabolism	l-Histidine; imidazole-4-acetate; methylimidazoleacetic acid	3/9	Imidazoleacetic acid, l-histidine	0.004	Down
Tyrosine metabolism	l-Noradrenaline; 3,4-dihydroxy-l-phenylalanine; l-tyrosine; 3-(4-hydroxyphenyl) pyruvate; pyruvate	5/33	**Pyruvic acid**, dihydroxyphenylalanine, 4-hydroxyphenylpyruvate	0.006	Both up and down
Citrate cycle (TCA cycle)	2-Oxoglutarate; succinate; citrate; pyruvate	4/20	**Pyruvic acid**, citric acid	0.023	Both up and down
*Saccharomyces cerevisiae*					
Glycine, serine, and threonine metabolism	l-Serine; l-cystathionine; glycine; l-threonine; d-glycerate; betaine; pyruvate	7/30	Cystathionine; betaine, d-Glycerate	0.004	Down
Tyrosine metabolism	l-Noradrenaline; 3,4-dihydroxy-l-phenylalanine; l-tyrosine; 3-(4-hydroxyphenyl) pyruvate; pyruvate	5/33	Dihydroxyphenylalanine, 4-hydroxyphenylpyruvate, norepinephrine	0.005	Down

^a^Over-accumulated metabolites appear in bold type, and down-accumulated metabolites appear in regular type.

## Discussion

Research on microbial supplementation of the black soldier fly diet has gained attention for its potential in waste bioconversion, leading to increased larval biomass and higher lipid and protein content (Yu et al. 2011, Abduh et al. 2017, Rehman et al. 2019, Somroo et al. 2019, Kooienga et al. 2020, Mazza et al. 2020, Wong et al. 2020, Franks et al. 2021, Elhag et al. 2022, Liew et al. 2022, Waters 2022). Nevertheless, no study has examined the nutritional content of these microbes. In this study, we focused on analyzing the amino acid content of different fungi related to the black soldier fly. Fungi are renowned for being rich sources of protein, establishing them as nutritional powerhouses with abundant amino acids and the capacity to supply B vitamins (Podpora et al. 2016). In the present study, *C. tropicalis* and *S. cerevisiae* had a protein content of 40% (dry matter), while previous reports indicate protein contents in the order of 50%–60% (Podpora et al. 2016, Mani et al. 2023a). Among the amino acids, leucine, lysine, arginine, asparagine, aspartic acid, glutamic acid, or glutamine and alanine were present in the tested fungi at over 3% (fungal dry weight); however, the lack of increase in amino acids in the black soldier fly larval hemolymph indicates their limited contribution, which requires further analysis in the future. As previously reported (Franks et al. 2021, Mani et al. 2023a), our study confirmed that supplementing the fungi, *P. kudriavzevii* and *S. cerevisiae*, led to increased larval body weight compared to the control. However, it is not clear whether this effect was due to direct consumption of the fungi by the insect or consumption of the metabolites extracted by the fungi in the insect gut.

The pathway enrichment analysis revealed that supplementation of *C. tropicalis* to the substrate increased the presence of metabolites related to aminoacyl-tRNA biosynthesis and glycerophospholipid metabolism pathways in black soldier fly larvae. Aminoacyl-tRNA synthesis is crucial for protein synthesis; thus, the observed over- and down-accumulation of metabolites in this pathway indicates that *C. tropicalis* may alter protein synthesis (Ibba and Dieter 2000). Interestingly, there was an increase in valine in this pathway, although the proportion of this amino acid was not higher in *C. tropicalis*. Over-accumulation of glycerophospholipid metabolites (such as acetylcholine and phosphocholine) in the *C. tropicalis* treatment suggests that *C. tropicalis* plays a crucial role in various cellular processes during larval growth of black soldier fly (Carman and Zeimetz 1996). Similarly, the down-accumulation of metabolites related to the histidine metabolism pathway in *C. tropicalis* and *P. kudriavzevii* treatments, compared to the water treatment, suggests their potential to influence protein synthesis and enzyme activity (Zhou et al. 2020). Furthermore, both *P. kudriavzevii* and *S. cerevisiae* treatments mostly reduced the abundance of metabolites related to tyrosine metabolism, consistent with previous research (Mani et al. 2023a). This may indicate that the tyrosine metabolism pathway increases in the larval body at the expense of related metabolites from the larval hemolymph. This pathway plays a crucial role in the production of neurotransmitter-related metabolites and melanin pigments in black soldier fly larvae, as reported previously (Fuchs et al. 2014, Sasaki, 2016).

In our study, we made interesting observations regarding pyridoxal and arginine, which did not show significant enrichment in the pathway analysis. Pyridoxal, a metabolite involved in B-6 vitamin metabolism, was found to be upregulated in all treatments compared to the control. This finding aligns with previous studies (Gusteleva, 1975, Salem et al. 2014, Gu et al. 2022, Mani et al. 2023a) demonstrating that insects lack the biosynthetic machinery to produce B vitamins, suggesting that *C. tropicalis* may serve as a source of B vitamins for black soldier fly larvae. In contrast, arginine exhibited downregulation in the *C. tropicalis* treatment compared to the control. Arginine is an important amino acid, playing a crucial role in pathogen–host interactions (Ding et al. 2021). Despite all 3 fungi having approximately 3% arginine content, only the *C. tropicalis* treatment had an effect on arginine levels in the black soldier fly larval hemolymph. Arginine may have been consumed by the fungus as a carbon source (Ding et al. 2021); however, this requires further investigation. These findings are consistent with other experimental evidence demonstrating that supplementation with *C. tropicalis* significantly increases the accumulation of metabolites from B-6 vitamin metabolism while decreasing the production of metabolites from arginine biosynthesis (Mani et al. 2023a). Further research is required to unravel the underlying mechanisms and identify the specific metabolites involved in this interaction between fungi and black soldier fly using functional metabolomics. Interestingly, hypoxanthine was found to be over-accumulated in a previous study (Mani et al. 2023a), but down-accumulated in the present study. This discrepancy may be attributed to the accumulation of metabolites in specific tissues, highlighting the difference between whole larval and hemolymph metabolomics analysis. Usually, fungi produce citric acid (Borekci et al. 2021), but this study found, in contrast, that citric acid was downregulated in the *P. kudriavzevii* treatment. Further study is required to understand why fungi alter metabolites within the citrate cycle, which is involved in alanine, aspartate, and glutamate metabolism and provides energy to the host (Zhou et al. 2020). Remarkably, the over-accumulation of quinolinic acid, a pyridinecarboxylic acid, in the *C. tropicalis* treatment suggests its potential derivation from tryptophan in the ingested diet, as reported for *Spodoptera littoralis* (Pesek et al. 2015). This implies that quinolinic acid may be derived from the fungal-supplemented diet.

We conclude that *P. kudriavzevii* exhibits potentially higher amounts of necessary amino acids, while *C. tropicalis* and *S. cerevisiae* exhibit distinct effects on larval hemolymph metabolites. These findings emphasize the potential of *P. kudriavzevii* as a fungal source for enhancing the nutritional value and body weight of black soldier fly larvae as a sustainable protein source. To further enhance our understanding, future studies could explore the transcriptomics of black soldier fly larvae and the metabolomics of both larvae and compost following treatment with various fungi. Such investigations would provide additional evidence for determining whether the black soldier fly directly ingests fungi or whether the fungi affect the larval diet and subsequently provide essential nutrients to black soldier fly larvae. These comprehensive analyses would deepen our knowledge of fungal–metabolite interactions and their implications for optimizing black soldier fly rearing and waste recycling practices for further application in other systems of insect rearing, as seen with *Tenebrio molitor* (Coleoptera: Tenebrionidae) (Rizou et al. 2022).

## Supplementary Material

ieae050_suppl_Supplementary_Table_S1

ieae050_suppl_Supplementary_Table_S2

ieae050_suppl_Supplementary_Table_S3
